# Biofluid‐based biomarker discovery using brain biopsy data as the reference standard

**DOI:** 10.1002/alz70856_097564

**Published:** 2025-12-24

**Authors:** Henrik Zetterberg

**Affiliations:** ^1^ University of Gothenburg, Mölndal, Gothenburg, Sweden

## Abstract

**Background:**

Shunt surgery for the treatment of normal pressure hydrocephalus (NPH) offers a unique opportunity for brain biopsy. The presentation describes biomarker discovery through tandem mass tag (TMT) proteomics of cerebrospinal fluid (CSF) samples where the included individuals were classified for Alzheimer's disease (AD) neuropathology according to the amyloid, tau and neurodegeneration (ATN) criteria using brain biopsy data.

**Method:**

Using liquid chromatography coupled with mass‐spectrometry (LC‐MS), we analyzed TMT multiplex CSF samples from NPH patients classified as positive or negative for AD neuropathology on the basis of immunohistochemistry of brain biopsy samples (*n* = 73). Validation cohorts were the Translational Biomarkers in Aging and Dementia (*n* = 134) and European Medical Information Framework (*n* = 467) sample sets.

**Result:**

We quantified over 1500 CSF proteins across the AD continuum in three independent cohorts, finely staged by Aβ/tau positron emission tomography (PET), fluid biomarkers, or brain biopsy. We identified clusters of proteins robustly correlating in all cohorts that sequentially changed with AD progression. Neurodegeneration‐related proteins (e.g., YWHAG, PPIA) were strongly associated with fluid, imaging and biopsy neurodegeneration biomarkers, increasing in parallel with endo‐lysosomal proteins. Metabolic proteins increased mid‐stage, followed by decreases in synaptic/membrane proteins. Our study identified potential PET imaging proxies (SMOC1, YWHAE, CNN3), and highlighted the dynamic fluctuations of the CSF proteome over the disease course, identifying candidate biomarkers for disease staging beyond Aβ and tau.

**Conclusion:**

Our study confirms and suggests novel biomarker candidates associated with ATN pathology and shows the temporal profile of different groups of CSF proteins across the AD continuum in three independent cohorts. The novel protein biomarkers could be useful for AD staging beyond the ATN framework.

